# Diabetes and Hypertension Consistently Predict the Presence and Extent of Coronary Artery Calcification in Symptomatic Patients: A Systematic Review and Meta-Analysis

**DOI:** 10.3390/ijms17091481

**Published:** 2016-09-06

**Authors:** Rachel Nicoll, Ying Zhao, Pranvera Ibrahimi, Gunilla Olivecrona, Michael Henein

**Affiliations:** 1 Department of Public Health and Clinical Medicine, Umea University and Heart Centre, Umea SE-901-87, Sweden; rachelnicoll25@gmail.com (R.N.); pranvera_i86@hotmail.com (P.I.); 2 Department of Ultrasound, Beijing Anzhen Hospital, Capital Medical University, Beijing 100029, China; yingzhaoecho@163.com; 3 Department of Medical Biosciences, Umea University, Umea SE-901-87, Sweden; gunilla.olivecrona@medbio.umu.se

**Keywords:** meta-analysis, systematic review, coronary calcification, risk factors

## Abstract

Background: The relationship of conventional cardiovascular risk factors (age, gender, ethnicity, diabetes, dyslipidaemia, hypertension, obesity, exercise, and the number of risk factors) to coronary artery calcification (CAC) presence and extent has never before been assessed in a systematic review and meta-analysis. Methods: We included only English language studies that assessed at least three conventional risk factors apart from age, gender, and ethnicity, but excluded studies in which all patients had another confirmed condition such as renal disease. Results: In total, 10 studies, comprising 15,769 patients, were investigated in the systematic review and seven studies, comprising 12,682 patients, were included in the meta-analysis, which demonstrated the importance of diabetes and hypertension as predictors of CAC presence and extent, with age also predicting CAC presence. Male gender, dyslipidaemia, family history of coronary artery disease, obesity, and smoking were overall not predictive of either CAC presence or extent, despite dyslipidaemia being a key risk factor for coronary artery disease (CAD). Conclusion: Diabetes and hypertension consistently predict the presence and extent of CAC in symptomatic patients.

## 1. Introduction

The presence of conventional cardiovascular (CV) risk factors (hypertension, diabetes, dyslipidaemia, smoking, obesity, and family history of coronary artery disease) have been shown to predict the 10-year coronary event risk [1,2,3]. In patients at intermediate risk, coronary artery calcification (CAC) is described as a subclinical form of atherosclerosis, often occurring as calcified atheroma or spotty calcification within a lipid core. Its measurement is commonly used clinically to avoid an invasive angiogram or as a marker for atherosclerosis in studies [4]. Similarly, the conventional CV risk factors may be used clinically to assess the likelihood of coronary calcification. Since both the CV risk factors and the presence and extent of CAC are predictive of coronary event risk [5,6], we investigated for the first time, in a systematic review and meta-analysis, whether conventional risk factors were also predictive of CAC presence, extent or progression in symptomatic patients. We hoped this would also throw more light on the phenomenon of coronary calcification, which in severe form could represent a clinical challenge as patients tend not to respond to conventional anti-anginal therapy [7]. Furthermore, as there is currently no specific treatment for arterial calcification, with atherosclerosis therapy such as statins and vasodilators having little effect [8], we hope that identifying specific predictive risk factors may point the way towards a remedy which could help prevent or even slow the process of coronary calcification.

## 2. Methods

The methodology for this systematic review and meta-analysis conforms to the Preferred Reporting Items for Systematic Reviews and Meta-Analyses statement [9].

### 2.1. Information Search and Data Collection

We systematically searched electronic databases (PubMed, MEDLINE, EMBASE, and Cochrane Centre Register) for observational human studies, assessing CAC and conventional CV risk factors. Articles were selected if the title or abstract indicated that the paper analysed original associations between CAC and CV risk factors using different combinations of the Medical Subject Headings (MeSH): “coronary calcification” or “coronary calcium” and “risk factors”, “hypertension”, “dyslipidaemia”, “hyperlipidaemia obesity”, “diabetes”, “smoking”, “family history”, “exercise” or “physical activity”. No date limit was applied to article selection. Since computed tomographic scanning for CAC was first introduced in the early 1990s, the study dates range from then to the present date. Two researchers performed the literature search, study selection and data extraction independently of each other, with the results placed in a spreadsheet; disagreements were resolved by discussion between the two researchers and a third adjudicated in case of disagreement. The selected reports were manually searched and other relevant articles, obtained from the reference lists, were retrieved. We also performed a quality assessment of each study included in the meta-analysis.

### 2.2. Study Eligibility Criteria

Any clinical studies that reported the presence, extent, new development or progression of CAC, assessed by electron beam computed tomography (EBCT), multi-detector computed tomography (MDCT), or coronary angiography, were eligible, regardless of whether or not the study objective was an assessment of the association of risk factors with CAC.

Study inclusion criteria were:

(a) English language articles published in peer-reviewed journals;
(b) In addition to age, gender, and ethnicity (where applicable), the study must include assessment of at least three risk factors out of dyslipidaemia, hypertension, obesity, diabetes, smoking, family history of premature CVD, or exercise. In some cases surrogate markers were used to indicate the presence of a risk factor, such as elevated low density lipoprotein (LDL) cholesterol to indicate hyperlipidaemia or elevated systolic blood pressure (SBP) consistent with hypertension. Any differences in risk factor criteria between studies are discussed in the narrative;
(c) The ability of risk factors to predict CAC presence, extent, new development, or progression must be displayed in a table rather than as a narrative. This criterion was included because narrative results in some studies did not adequately reflect the tabular results, for example, where a risk factor shown as significant in a table was not mentioned in the narrative. Studies also varied in their treatment of a *p*-value of 0.05, with some taking it as borderline and others as significant but the exact *p*-value may not be shown in the narrative; for our purposes only *p* < 0.05 is taken as significant,
(d) The patients must be symptomatic (complaining of chest pain or any other typical or atypical angina symptoms); and
(e) For the systematic review only, the study results must show risk factors as multivariate predictors of CAC presence, extent, or progression.

Study exclusion criteria were:

Those involving patients with a specific diagnosis, such as Type 1 diabetes or renal disease, which had no healthy control group.
There were no specified requirements for the control groups, where applicable.

### 2.3. Statistical Analysis

The data was extracted from each study and analysed using the Revman software 5.3 (Copenhagen, Denmark: The Nordic Cochrane Centre, The Cochrane Collaboration, 2014). The publication bias was tested using Egger’s regression interception test and funnel plot by comprehensive meta-analysis software. The unadjusted odds ratios (ORs) of each risk factors were estimated from the exposure distributions for CAC presence or absence. The ORs and 95% confidence intervals (CIs) were converted into Log OR and standard error (SE) using the calculator and the Revman software in order to obtain the forest plots for each risk factor. The statistical heterogeneity was evaluated using the I^2^ statistical test. When the I^2^ was greater than 50%, the analysis was considered significantly heterogeneous and the random effect model was applied. When the I^2^ was less than 50%, the analysis was considered not heterogeneous and the fixed effect meta-analysis model was applied. A *p*-value of <0.05 was regarded as significant.

## 3. Results

### 3.1. Data Extraction

**Figure 1:**
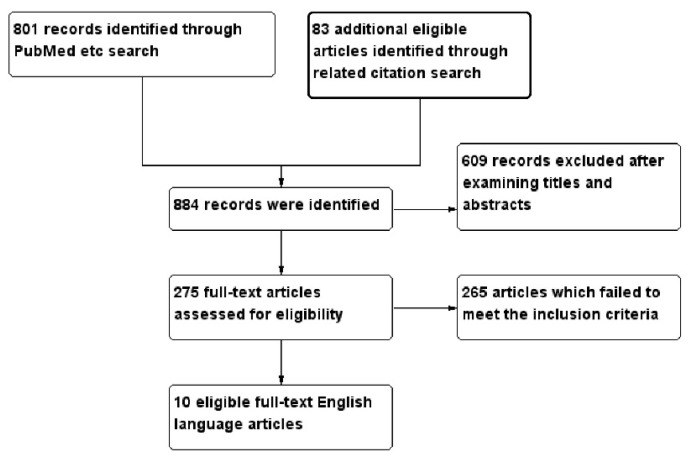
Flowchart showing selection of eligible studies.

A total of 884 studies were identified. After exclusion of duplicates and review of the retrieved papers for the above criteria (the selection process is shown in Figure 1), 10 studies comprising 15,769 symptomatic patients [10,11,12,13,14,15,16,17,18,19] were eligible for inclusion in the systematic review, while seven studies comprising 12,682 patients [10,11,12,14,16,17,18] were eligible for inclusion in the meta-analysis. All are listed in Table 1. A risk factor was included in the meta-analysis when at least three papers had provided data on that risk factor. The papers were then divided according to CAC assessment type (i.e., CAC presence, extent or progression). One study, Lai et al. [10], assessed both CAC presence and extent and is, consequently, shown twice in both the systematic review and meta-analysis, while studies by Mayer et al. [11] and Mitsutake et al. [12] could be used for both CAC presence and extent in the meta-analysis but were used only for CAC extent in the systematic review.

CAC presence was defined as any CAC score >0. CAC extent was defined as the amount of the CAC score in studies which did not use a CAC score threshold or, in studies which did use a CAC score threshold, CAC extent was defined as any CAC score >100 compared to CAC = 0; any study with a CAC score threshold ≤100 was taken as a study of CAC presence. Similarly in angiographic studies, where moderate/severe calcification was compared with mild/no calcification, this was also taken as a study of CAC presence. CAC progression was defined as an increase in the CAC score over time.

Ten studies fitted our inclusion criteria [10,11,12,13,14,15,16,17,18,19], comprising 15,769 symptomatic patients, as outlined in Table 1. The number of patients ranged from 114 in Maragiannis et al. [17], to 1560 in Greif et al. [15], with one study of CAC presence by Kovacic et al. [16] comprising 9993 patients; due to the large numbers in this study, it will be separately mentioned in the analysis unless its results conform to those of all other studies. Three studies had a solely Japanese population [12,13,19], two studies were Chinese [10,18], while one was Turkish [14]. All studies were mixed gender, except for Lai et al. [10] and Mayer et al. [11], which investigated exclusively male patients. One study, Greif et al. [15], separately investigated males and females and, consequently, this was treated as two separate studies [15] in the analysis. All studies investigated a wide age range except Lai et al. [10], whose patients were aged ≥65. All patients in the study by Kovacic et al. [16] had coronary stenosis ≥60%, while those in the study by Mayer et al. [11] had CAD and a close relative who had suffered a myocardial infarction before the age of 60 years. Eight of the 10 studies had CAC assessed by CT scanner, either 16- or 64-slice, but the remaining two were investigated angiographically. Kovacic et al. [16] assessed the extent of CAC on the stenotic lesion undergoing percutaneous coronary intervention (PCI), with CAC being graded as none, mild, moderate or severe, while Mayer et al. [11] assessed the CAC observed in the coronary vessels as none, mild-moderate, or severe. Kovacic et al. [16] assessed predictors for calcification as moderate-severe calcification compared to no calcification; we have included this in the analysis as a study of CAC presence rather than CAC extent.

#### 3.1.1. Systematic Review

We analysed the following numbers of studies in each category:
CAC presence cross-sectionalsix studies12,830 patientsCAC extent cross-sectionalfour studies2986 patientsCAC progressionone study164 patientsTotal symptomatic patients15,980 patients

However, Lai et al. [10], with 211 patients, was included in both CAC presence and extent.

#### 3.1.2. Meta-Analyses

We analysed the following numbers of studies in each category, with three studies providing data for both CAC presence and extent:
CAC presence cross-sectionalseven studies12,682 patientsCAC extent cross-sectionalthree studies1623 patients

The unadjusted ORs of each risk factors were estimated from the exposure distributions for CAC presence or absence [20,21], with the exception of the ORs from the study by Kovacic et al. [16], which directly showed the univariate ORs in the results. Since age was a continuous parameter, the OR for age was pooled from multivariate results. For the remaining risk factors, few papers provided the multivariate ORs, so consequently the pooled ORs from multivariate results were not analysed. In the three papers which provided the ORs for both CAC presence and extent [10,11,12], we extracted the exposure distributions for moderate and severe CAC and combined them as CAC presence. In these three papers, the ORs for the comparison between mild to moderate CAC and zero CAC and the comparison between severe CAC and zero CAC were pooled from the exposure distribution separately to assess the risk factors which predicted CAC extent.

### 3.2. Systematic Review

The papers were then analysed by age, gender, ethnicity, diabetes, dyslipidaemia, family history, hypertension, obesity, and smoking (Table 2). In none of the studies was physical activity assessed. In several studies the definition of the risk factor comprised multiple components for example, dyslipidaemia could include any of elevated total, LDL cholesterol or the total/HDL cholesterol ratio, or decreased HDL cholesterol. For the purposes of analysis for the systematic review, a risk factor was assessed if any one of its components was present (so dyslipidaemia was predictive if only LDL cholesterol was elevated and all other components were in normal range). Analysis of the precise risk factor components is also provided in each relevant section, where any modifying effect of age is also considered.

Age: There are five studies showing that age is predictive of CAC presence [14,15,16,17,18], compared to one which is not predictive [10], although in this study all patients were as ≥65. Three studies showed that age was predictive of CAC extent [11,12,13] and the same study of patients aged ≥65 found age not to be predictive [10]. The only study of CAC progression found that age was not predictive [19].

Male gender: two studies of CAC presence showed that gender was not predictive [14,16], including the angiographic study of 9993 patients [16], although in two studies of CAC presence [17,18] and two studies of CAC extent it was predictive [12,13].

Ethnicity: The study of 9993 patients was the only one to consider ethnicity and this found that being white was predictive of CAC presence [16].

Diabetes mellitus: The two Greif et al. studies [15] and the angiographic study of 9993 patients [16] found diabetes to be predictive of CAC presence, although four smaller studies showed that it is not [10,14,17,18]. Although one study of 1363 patients found diabetes to be predictive of CAC extent [13], three studies with a total of 1623 patients showed that it was not predictive [10,11,12]. The only study of CAC progression showed that diabetes was predictive [19].

When the studies are analysed by markers for diabetes:

- Blood glucose was not predictive of CAC presence [14] or extent [12].
- Insulin was not predictive in one study of CAC presence and extent [10].
- HbA1c was not predictive of CAC presence [15,18] or extent [11,12], although it did show predictive ability for CAC progression [19].
- Oral hypoglycaemic medication was not predictive in one study of CAC presence and extent [10].
- Homeostatic Model Assessment-Insulin Resistance (HOMA-IR) was not predictive of CAC presence [15].

Dyslipidaemia: Thee studies showed that dyslipidaemia was predictive of CAC presence [15,18], while four studies, including the study of 9333 patients, shows that it is not predictive [10,14,16,17]. For CAC extent, however, one study showed that dyslipidaemia was predictive [11] but three studies, with more than twice as many patients, found it not to be predictive [10,12,13]. The one study of CAC progression [19] showed that it was not predictive.

When the studies are analysed by markers and biomarkers for dyslipidaemia:

- Elevated LDL cholesterol was not predictive in all four studies of CAC presence [14,15,16] and in two studies of CAC extent [11,12].
- Elevated total cholesterol was not predictive in two studies of CAC presence [14,18], two studies of CAC extent [11,12], and one study of CAC progression [19].
- Decreased HDL was not predictive in three studies of CAC presence [14,15] and one study of CAC extent [12], but was predictive of severe CAC extent in one study [11].
- Lipid-lowering medication was predictive in three studies of CAC presence [15,18], but was not predictive in one study of CAC presence [10] and two studies of CAC extent [10,12].

Hypertension: Although two studies, including the study of 9993 patients, [16,17] found that hypertension was predictive of CAC presence, the remaining studies showed that it had no predictive ability for CAC presence [10,13,15,18]. Three studies of CAC extent found that it was predictive [11,12,13], but one study found it not to be predictive [10]; the one study of CAC progression was also predictive [19].

When the studies are analysed by markers for hypertension:

- Systolic blood pressure (SBP) was not predictive in three studies of CAC presence [10,15] and two studies of CAC extent [10,12] but one angiographic study showed it was predictive of severe CAC extent [11]. SBP was also predictive of CAC progression [19].
- Diastolic blood pressure (DBP) was not predictive in three studies of CAC presence [10,15] and three studies of CAC extent [10,11,12].
- Antihypertensive medications were not predictive in four studies of CAC presence [10,15,18] and one of CAC extent [10].
- Pulse pressure was not predictive in one study of CAC extent [11] and the study of CAC progression [19].

Family history of premature CHD: The only study to assess predictive ability for CAC presence was the study of 9993 patients [16], which found it was not predictive. Among those investigating CAC extent, one was predictive, with 877 patients, [11] but another was not, with 535 patients [12]. The one predictive study of CAC extent was angiographic and investigated a family history of CAC since the population was preselected to comprise males with a family history of CAD [11].

Obesity: Five studies found no ability for obesity to predict CAC presence [10,15,17,18], although the large study by Kovacic et al. found that there was an inverse predictive ability between obesity and CAC presence, making obesity protective against CAC [16]. No study of CAC extent, found that obesity was predictive [10,11,12]. The only study of CAC progression did not find obesity to be predictive [19].

When the studies are analysed by markers for obesity:

- Body mass index (BMI) was inversely predictive in the angiographic study of CAC presence involving 9993 patients [16], but not in a further three studies of CAC presence [10,17,18] and one of CAC extent [10]. BMI was not predictive in one study of CAC progression [19].
- Weight was not predictive in one study of CAC extent [16].

Smoking: With respect to CAC presence, only one small study found it to be predictive [14], while the remainder, including the study of 9993 patients, found that smoking was not predictive of CAC presence [10,15,16,18]. None of the four studies of CAC extent [10,11,12,13] found smoking to be predictive.

When the studies are analysed by markers for smoking, current smoking was not predictive in two studies of CAC presence [15].

### 3.3. Meta-Analysis

Out of the ten papers that were eligible for the systematic review [10,11,12,13,14,15,16,17,18,19], seven were also suitable for the meta-analysis: Lai et al. [10], Mayer et al. [11], Mitsutake et al. [12], Atar et al. [14], Kovacic et al. [16], Maragiannis et al. [17], and Qing et al. [18].

The meta-analysis investigated the predictive ability of age, male gender, diabetes, dyslipidaemia, hypertension, and smoking for CAC presence and extent (Supplementary Materials, Figure S1). It was not possible to include ethnicity, obesity, exercise, or number of risk factors, although family history of CAD was not predictive; since no other study assessed these two risk factors, they have not been entered in the meta-analysis. As mentioned above, data from three studies investigating CAC extent in the systematic review (Lai et al. [10], Mayer et al. [11], and Mitsutake et al. [12]) have been re-analysed to identify potential risk factor predictors of CAC presence in the meta-analysis.

#### 3.3.1. Predictors of CAC Presence

Table 3 gives the pooled results from the meta-analysis. The predictors of CAC presence in order of importance were hypertension (OR = 1.71, *p* < 0.00001), male gender (OR = 1.47, *p* = 0.02), diabetes (OR = 1.34, *p* = 0.03), and age (OR = 1.07, *p* = 0.04). Smoking and dyslipidaemia were not predictive of CAC presence. The Egger’s regression interception test was not significant suggesting no significant publication bias (Table 3). Age, being a continuous variable, could not be entered into the Egger test. The funnel plots for each risk factor are provided in the supplementary Figure S2 and, similarly, show no publication bias.

Due to the disproportionately large number of patients in the study by Kovavic et al. [16], we repeated the meta-analysis after excluding this paper (shown in the supplementary data, Table S1). This slightly increased the ORs for hypertension to 1.89 (*p* < 0.00001), male gender to 1.74 (*p* < 0.00001), diabetes to 1.45 (*p* < 0.00001). Smoking and dyslipidaemia were still not significant.

#### 3.3.2. Predictors of CAC Extent

Only three studies (Lai et al. [10], Mayer et al. [11], and Mitsutake et al. [12]) analysed the predictors of CAC extent, among which Mayer et al. was an angiographic study classifying CAC as either “no calcification”, “mild to moderate calcification”, or “severe calcification”. Mitsutake et al. [12] used CAC scoring and classified the lowest group (taken to be CAC = 0) as a CAC score of 0–12, the mild-moderate group as a CAC score of 13–445, and the severe calcification group as a CAC score of >445, while Lai et al. [10] used a threshold CAC score of ≥400 The results are shown in Table 4.

The presence of mild-moderate CAC, compared with zero CAC, was independently predicted only by hypertension (OR 1.61, *p* < 0.0001), with diabetes, dyslipidaemia, and smoking proving not to be predictive of mild-moderate CAC. The presence of severe CAC, compared with zero CAC, was predicted by hypertension (OR 2.09, *p =* 0.01) and diabetes (OR 1.55, *p =* 0.005); dyslipidaemia and smoking were not independently predictive of severe CAC. It was not possible to analyse age or male gender as predictors of CAC extent.

A summary of the studies showing the predictive ability of the risk factors from the systematic review and meta-analysis are shown at Table 5. 

#### 3.3.3. Quality Assessment

We carried out a MINORS evaluation of the studies included in the meta-analysis, as shown at Table 6. The items are scored 0 (not reported), 1 (reported but inadequate), or 2 (reported and adequate), with the global ideal score being 16 for non-comparative studies. Most studies scored 2 for all parameters, except follow-up data and prospectivity, which were obviously not in the design for our case-control studies. These results were considered quite satisfactory.

## 4. Discussion

### 4.1. Findings

In the Systematic Review, age was strongly predictive of both CAC presence and extent, but not of CAC progression. The results for other risk factors for CAC presence are not as clear cut, largely due to the Kovacic et al. [16] study of 9993 patients, which overwhelmed the analysis. This study found that white ethnicity, diabetes, hypertension, and obesity were predictive of CAC presence, but not male gender, dyslipidaemia, family history, or smoking. These results do not necessarily accord with the totality of the studies, in which a broadly equal number showed that male gender, diabetes, and dyslipidaemia were predictive of CAC presence as not predictive. Only two studies (including Kovacic et al. [16]) found that hypertension was predictive of CAC presence, compared to five studies finding that it was not predictive, while only Kovacic et al. [16] found that obesity was predictive (albeit inversely), whereas five studies found that it was not predictive of CAC presence. Smoking was, overall, not predictive. No study of CAC presence, other than Kovacic et al. [16], assessed ethnicity (predictive) or family history of CAD (not predictive). With respect to CAC extent, male gender, hypertension, and possibly a family history of CAC were predictive, but diabetes, dyslipidaemia, obesity, and smoking were, overall, not predictive. For diabetes there were an almost equal amount of patient numbers in the three studies which found diabetes to be predictive as not predictive of CAC extent. In the one study of CAC progression, diabetes and hypertension were predictive, but not age, dyslipidaemia, or obesity.

Among the risk factor markers, only use of lipid-lowering medication and higher BMI were broadly predictive of CAC presence, with possibly decreased HDL and increased SBP being predictive of CAC extent, although these results were found in only one study. In the single study of CAC progression, HbA1c and SBP were predictive.

The meta-analysis included seven studies, rather than the ten in the systematic review, although two studies of CAC extent also provided sufficient statistical data to be used to assess CAC presence, while another study provided data for both CAC presence and extent. This analysis shows that hypertension followed by male gender, diabetes, and age were predictive of CAC presence, while smoking and dyslipidaemia were not predictive. For CAC extent, however, mild-moderate CAC was predicted by hypertension alone, whereas severe CAC was predicted by hypertension followed by diabetes. The MINORS scores were quite satisfactory for all included studies which adds to the strength of the data analysis.

### 4.2. Areas of Difference between Results from the Systematic Review and Meta-Analysis

The most striking difference between the results from the systematic review and the meta-analysis is the minimal importance of age as a predictor of CAC in the meta-analysis, whereas it is a consistent predictor of both CAC presence and extent in the systematic review. However, this may largely be accounted for, firstly, by the fact that age is a continuous variable and, secondly, that a different mix of studies of CAC presence were used for the systematic review and meta-analysis. We have previously shown the important predictive value of age in a large cohort of symptomatic patients [23].

With respect to CAC presence, there were no other clear predictive risk factors based on the numbers of studies but when considering numbers of patients then the Kovacic et al. [16] study of 9993 patients, which found that diabetes and hypertension were predictive, was broadly in agreement with the meta-analysis. In the systematic review, CAC extent was predicted by male gender and hypertension, whereas in the meta-analysis CAC extent was predicted by hypertension and diabetes; this can be explained by the different mix of studies between the two methods. The main limitation of the systematic review is its qualitative analysis with many contributory factors, such as the power of the study, the number of studies, and the number of patients. These limitations are overcome by the quantitative pooling of the meta-analysis.

### 4.3. Comparison with Other Studies

Although we have found that in symptomatic patients the predictive risk factors for CAC presence, extent, and progression are hypertension and diabetes, this is not the case in asymptomatic subjects where dyslipidaemia, smoking, obesity, and family history of CAD have also been shown to be predictive of CAC presence and progression in large population studies, such as the Multi-Ethnic Study of Atherosclerosis and Heinz Nixdorf Recall [24,25]. No systematic review or meta-analysis of risk factor predictors for CAC in asymptomatic subjects has been carried out. Although the two conditions, hypertension and diabetes, are different in their clinical presentation and means of treatment, their effect on the arterial wall seems to be phenotypically similar, suggesting a shared mechanism such as oxidative stress [26,27]. Arterial calcification represents segmental ossification which is known to be progressive even after controlling risk factors, thus suggesting a perpetual effect, through a biochemical and/or histopathological mechanism, of those risk factors rather than just a triggering effect that subsides with their optimum control. Nevertheless, there is no inherent reason why the conventional CV risk factors, which were identified as predictors of 10-year coronary event risk [28,29], should predict CAC presence or extent, merely because CAC can also predict the 10-year event risk [3].

Curiously, the expected predictive risk of dyslipidaemia did not feature strongly in either the systematic review or the meta-analysis. While some studies have shown that dyslipidaemia can be predictive of arterial calcification presence or extent in asymptomatic subjects, this is not always the case, previously seen in a systematic review and meta-analysis of predictors of breast arterial calcification which found no relationship with dyslipidaemia [30]. In addition, dyslipidaemia is a particularly Caucasian problem [31] and it may be that the high number of studies with a Chinese or Japanese population included in the meta-analysis has impacted the results. Nevertheless, we have previously found that lipid-lowering medication has no effect on reducing coronary or aortic valve calcification [32,33], while other studies have found that rather than the treatment group, it is the placebo group that has less calcium progression [22,34]. It may, however, be the case that by the time calcification is established, the association with dyslipidaemia has been lost.

### 4.4. Limitations

A number of limitations deserve mention. Firstly, although we attempted to identify and include all relevant studies, there will inevitably be some that we have overlooked. Secondly, our search was restricted to studies in English, so it may be possible that some studies in other languages have been missed. Thirdly, the studies included in this systematic review and meta-analysis varied in design, population (e.g., eligibility by age), definition and duration of risk factor, and year of publication. As expected, we observed considerable heterogeneity between studies, so it is arguable whether a summary estimate should be presented. However, our objective was not to provide this but rather to present a general approximation of the prevalence of these risk factors to facilitate the message. In particular, two studies assessed CAC angiographically, which is not sensitive to CAC detection, while the remainder used 16- or 64-slice CT scanning. Fourthly, analysis of studies of CAC presence was overwhelmed by the study of 9333 patients, while the next largest study had only 1560. Fifthly, the lack of standardisation of definitions of risk factors limits our ability to provide summary estimates and we had no information on the duration of risk factors, which might have impacted the analysis. Sixthly, we were confined to those risk factors commonly measured in a clinical setting and, inevitably, there are others which might have been relevant.

## 5. Conclusions

Our meta-analysis showed that hypertension followed by diabetes were the most important risk factors for prediction of CAC presence and extent, with age and male gender also showing predictive ability for CAC presence. The results from the systematic review were more equivocal, but the two forms of analysis were in general agreement that dyslipidaemia, obesity, and smoking were not predictive of CAC presence or extent. Irrespective of the mechanism for arterial endothelial damage, hypertension and diabetes seem to result in a common phenotypic arterial wall damage in the form of calcification. Finally, despite CAC and the conventional CV risk factors both being predictive of 10-year coronary event risk, only a few of the CV risk factors appear predictive of CAC.

## Figures and Tables

**Table 1:** Study characteristics.

CAC Measurement	Author, Year	Reference No.	Study Population CAC = 0 CAC > 0 or Total Population If No Data Provided		Mean Age (Years)	Means of CAC Assessment	Notable Patient Characteristics
CAC Extent	Lai et al., 2015 as above	[10]	91	120	71.1	64-slice GE scanner	Chinese ethnicity, males aged ≥ 65
	Mayer et al., 2007	[11]	333	544	59.7	Angiographic, CAC observed in the coronary vessels could be none, mild-moderate or severe.	Males
	Mitsutake et al., 2007	[12]	245	290	64	16- or 64-slice Toshiba CT scanner	Japanese ethnicity
	Tanaka et al., 2012	[13]	1363		68	64-slice Toshiba CT scanner	Japanese ethnicity
CAC Presence	Atar et al., 2013	[14]	382	60	53.6	64-slice Phillips CT scanner	Turkish ethnicity
	Greif et al., 2013	[15]	Males	1123	55.4	16-slice Siemens CT scanner	European ethnicity
			Females	437	63.2		
	Kovacic et al., 2012	[16]	8553	1440	66.6	Angiographic, CAC on stenotic lesion undergoing PCI, could be none, mild, moderate or severe.	All with coronary stenosis ≥ 60%
	Lai et al., 2015	[10]	91	120	71.1	64-slice GE scanner	Chinese ethnicity, males aged ≥ 65
	Maragiannis et al., 2015	[17]	65	49	56.1	16-slice Phillips CT scanner	US study
	Qing et al., 2015	[18]	146	364	56.0	64-slice GE CT scanner	Chinese ethnicity
CAC Progression	Okada et al., 2013	[19]	164 (all with CAC > 0)		68.7	64-slice Toshiba CT scanner	Japanese ethnicity

All studies of CAC presence and extent were case-control studies, while the one study of CAC progression (Okada et al. [19]) was a cohort study.

**Table 2:** Systematic Review: analysis of the number and type of studies investigating risk factors for CAC.

Risk Factors	CAC Presence		CAC Extent		CAC Progression	
	Predictive	Not Predictive	Predictive	Not Predictive	Predictive	Not Predictive
Age	5	1	3	1	0	1
Gender	2	2	2	0	0	0
Ethnicity	1	0	0	0	0	0
Diabetes	3	4	1	3	1	0
Dyslipidaemia	3	4	1	3	0	1
Hypertension	2	5	3	1	1	0
Family history	0	1	1	1	0	0
Obesity	1	5	0	3	0	1
Smoking	1	5	0	4	0	0

**Table 3:** Meta-analysis: pooled risk factors and their ORs predicting CAC presence.

Risk Factors	Pooled or (95% CI)	*p* for Overall Effect	Studies	Patient Numbers	Egger’s Test		
					Intercept	*t*-Value	*p*-Value
Age (years)	1.07 (1.00–1.04)	0.04	[10,14,18]	1163			
Male gender	1.47 (1.05–2.06)	0.02	[12,14,16,17,18]	11,594	2.29	2.42	0.09
Hypertension	1.71 (1.51–1.94)	<0.00001	[10,11,12,14,16,17,18]	12,682	0.94	0.78	0.47
Diabetes mellitus	1.34 (1.02–1.75)	0.03	[10,11,12,14,16,17,18]	12,682	0.81	0.83	0.44
Smoking	1.42 (0.90–2.22)	0.13	[10,11,12,14,16,17,18]	12,682	3.39	1.84	0.12
Dyslipidaemia	1.25 (0.81–1.94)	0.31	[10,12,16,17]	10,853	1.09	0.64	0.59

**Table 4:** Meta-analysis: pooled risk factors and their ORs predicting CAC extent.

Risk Factors	Mild to Moderate CAC or CACS 13-445 vs. CACS = 0		Severe CAC or CACS > 445 vs. CACS = 0		Patient Numbers
	OR	*p*-Value	OR	*p*-Value	
Hypertension	1.61 (1.28–2.03)	<0.0001	2.09 (1.09–4.03)	0.0100	1623
Diabetes mellitus	1.22 (0.93–1.60)	0.1600	1.55 (1.14–2.10)	0.0050	1623
Dyslipidaemia	0.75 (0.52–1.00)	0.1300	1.03 (0.65–1.63)	0.9000	746
Smoking	0.93 (0.72–1.20)	0.6000	1.07 (0.68–1.67)	0.7700	1623

CACS = Coronary artery calcification score; Studies used in CAC extent meta-analysis: Lai et al. [10], Mayer et al. [11], and Mitsutake et al. [12]. Lai et al. [10], a study using a threshold of >400, was included as severe CAC.

**Table 5:** Summary of studies showing risk factor predictive ability for CAC presence, extent, or progression.

Risk Factors	SYSTEMATIC REVIEW References			Meta-Analysis References	
	CAC Presence	CAC Extent	CAC Progression	CAC Presence	CAC Extent
Age	[10,14,15,16,17,18]	10, 11–13	19	[10,14,18]	Not assessed
Male gender	[14,16,17,18]	12, 13	Not assessed	[12,14,16,17,18]	Not assessed
Ethnicity	[16]	Not assessed	Not assessed	Not assessed	Not assessed
Diabetes	[10,14,15,16,17,18]	13–10	19	[10,11,12,14,16,17,18]	10; 11
Dyslipidaemia	[10,14,15,16,17,18]	13–10	19	[10,12,16,17]	10; 11
Hypertension	[10,13,15,16,17,18]	13–10	19	[10,11,12,14,16,17,18]	10; 11
Family history	[16]	11,12	Not assessed	Not assessed	Not assessed
Obesity	[10,15,16,17,18]	12–10	19	Not assessed	Not assessed
Smoking	[10,14,15,16,18]	13–10	Not assessed	[10,11,12,14,16,17,18]	10; 11

Reference key: [10]: Lai et al., 221 Chinese males aged ≥65; [11]: Mayer et al., 877 males with CAD, angiographic study; [12]: Mitsutake et al., 535 patients, Japanese ethnicity; [13]: Tanaka et al., 1363 patients, Japanese ethnicity; [14]: Atar et al., 442 patients, Turkish ethnicity; [15]: Greif et al., 1123 males, European ethnicity; [15]: Greif et al., 437 females, European ethnicity; [16]: Kovacic et al., 9993 patients, angiographic study; [17]: Maragiannis et al., 114 patients, US study; [18]: Qing et al., 510 patients, Chinese ethnicity; [19]: Okada et al., 164 patients with CAC, Japanese ethnicity.

**Table 6:** Quality assessment of studies included in the meta-analysis.

Study	Clearly Stated Aim	Consecutive Patients Inclusion	Prospective Collection of Data	Endpoints Appropriate	Unbiased Assessment of the Study Endpoint	Follow-up Period Appropriate to the Aim of the Study	Loss to Follow up Less than 5%	Prospective Calculation of the Study Size	Total Score
Atar et al., 2013 [14]	2	2	2	2	2	0	0	0	10
Greif et al., 2013 [15]	2	2	2	2	2	0	0	0	10
Kovacic et al., 2012 [16]	1	2	0	1	2	0	0	0	6
Mayer et al., 2007 [11]	2	2	2	2	2	0	0	0	10
Mitsutake et al., 2007 [12]	2	2	2	2	2	0	0	0	10
Okada et al., 2013 [19]	2	2	2	2	2	2	1	0	13
Tanaka et al., 2012 [13]	2	2	2	2	2	0	0	0	10
Lai et al., 2015 [10]	2	2	2	2	2	0	0	0	10
Maragiannis et al., 2015 [17]	2	1	2	2	2	0	0	0	9
Qing et al., 2015 [18]	2	2	2	2	2	0	0	0	10

Evaluation of meta-analysis studies using the Methodological Index for Non-Randomized Studies (MINORS) [22]. Elements are scored 0 (not reported), 1 (reported but inadequate), or 2 (reported and adequate).

## Supplementary Materials

Supplementary materials can be found at www.mdpi.com/1422-0067/17/9/1481/s1.
