# Supplementary material for: Diabetes and Hypertension Consistently Predict the Presence and Extent of Coronary Artery Calcification in Symptomatic Patients: A Systematic Review and Meta-Analysis
Source: Int J Mol Sci. 2016 Sep 6;17(9):1481. doi: 10.3390/ijms17091481 (PMC5037759; doi:10.3390/ijms17091481)
Supplement: Supplementary file 1 [file ijms-17-01481-s001.pdf]

# Supplementary Materials: Diabetes and Hypertension Consistently Predict the Presence and Extent of Coronary Artery Calcification in Symptomatic Patients: A Systematic Review and Meta-Analysis

**Rachel Nicoll, Ying Zhao, Pranvera Ibrahimi, Gunilla Olivecrona and Michael Henein**

**Table S1.** Meta-analysis: Pooled risk factors and their ORs predicting CAC presence excluding Kovacic et al.

| Risk Factors | Pooled OR (95% CI) | *P* for Overall Effect | Studies | Patient Numbers | Egger's Test | | |
|---|---|---|---|---|---|---|---|
| | | | | | Intercept | *t* Value | *p*-Value |
| Age (years) | 1.07 (1.00–1.04) | 0.04 | [10,14,18] | 1163 | - | - | - |
| Male gender (yes/no) | 1.74 (1.38–2.20) | <0.00001 | [12,14,16–18] | 11,594 | −0.80 | 0.92 | 0.46 |
| Hypertension (yes/no) | 1.89 (1.59–2.23) | <0.00001 | [10–12,14,16–18] | 12,682 | −0.21 | 0.12 | 0.91 |
| Diabetes mellitus (yes/no) | 1.45 (1.18–1.78) | <0.00001 | [10–12,14,16-18] | 12,682 | −0.81 | 0.37 | 0.73 |
| Smoking (yes/no) | 1.53 (0.82–2.86) | 0.18 | [10–12,14,16–18] | 12,682 | 5.89 | 1.35 | 0.25 |
| Dyslipidaemia (yes/no) | 1.60 (0.56–4.51) | 0.38 | [10,12,16,17] | 10,853 | 2.87 | 1.39 | 0.39 |

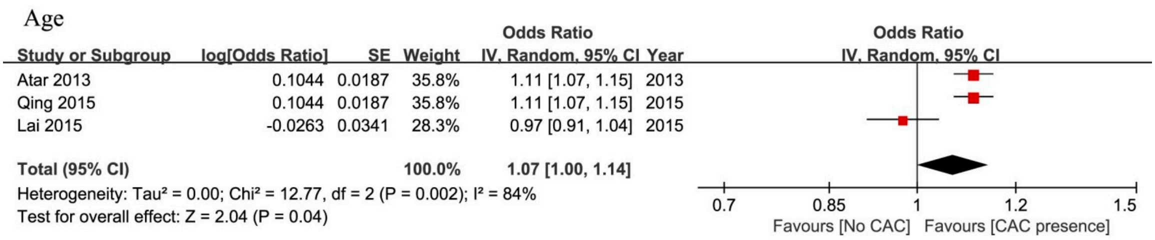


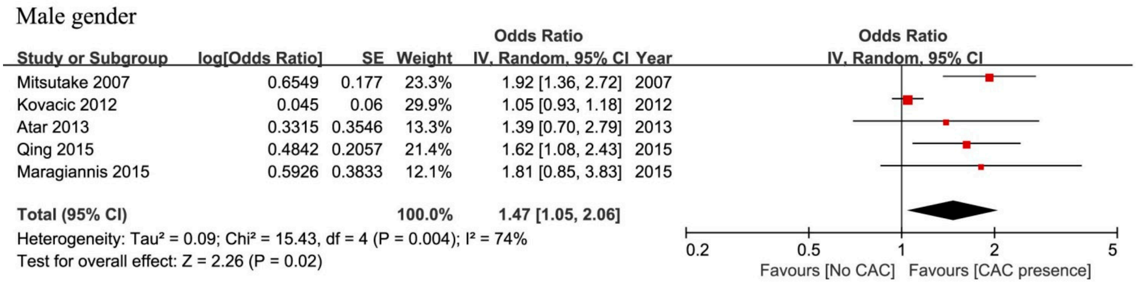


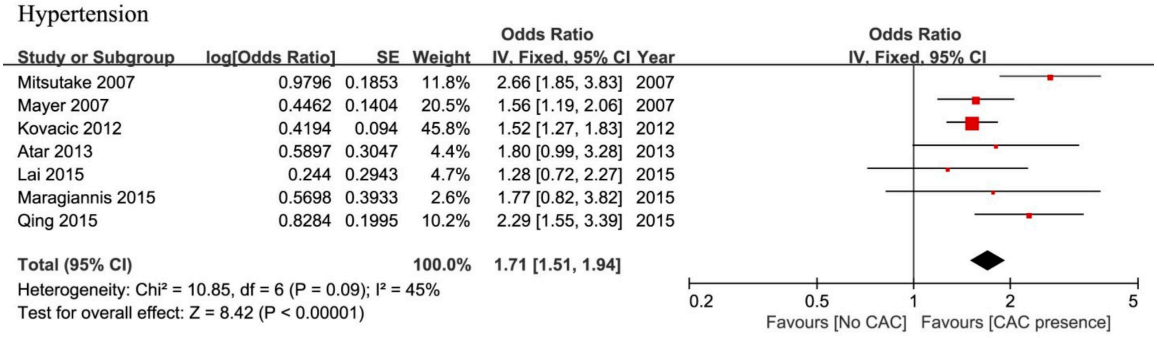


**Figure S1.** *Cont.*

Diabetes melitus

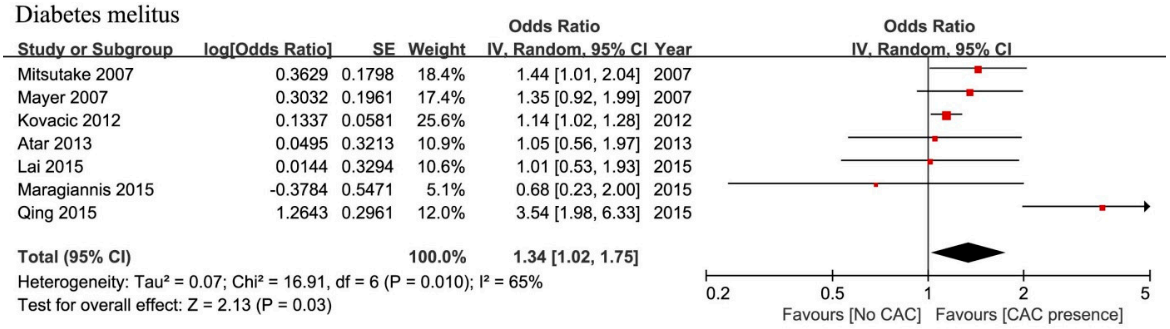

| Study or Subgroup | log[Odds Ratio] | SE | Weight | Odds Ratio IV, Random, 95% CI | Year |
|---|---|---|---|---|---|
| Mitsutake 2007 | 0.3629 | 0.1798 | 18.4% | 1.44 [1.01, 2.04] | 2007 |
| Mayer 2007 | 0.3032 | 0.1961 | 17.4% | 1.35 [0.92, 1.99] | 2007 |
| Kovacic 2012 | 0.1337 | 0.0581 | 25.6% | 1.14 [1.02, 1.28] | 2012 |
| Atar 2013 | 0.0495 | 0.3213 | 10.9% | 1.05 [0.56, 1.97] | 2013 |
| Lai 2015 | 0.0144 | 0.3294 | 10.6% | 1.01 [0.53, 1.93] | 2015 |
| Maragiannis 2015 | -0.3784 | 0.5471 | 5.1% | 0.68 [0.23, 2.00] | 2015 |
| Qing 2015 | 1.2643 | 0.2961 | 12.0% | 3.54 [1.98, 6.33] | 2015 |
| **Total (95% CI)** | | | **100.0%** | **1.34 [1.02, 1.75]** | |

Heterogeneity: Tau² = 0.07; Chi² = 16.91, df = 6 (P = 0.010); I² = 65%
Test for overall effect: Z = 2.13 (P = 0.03)



Smoking

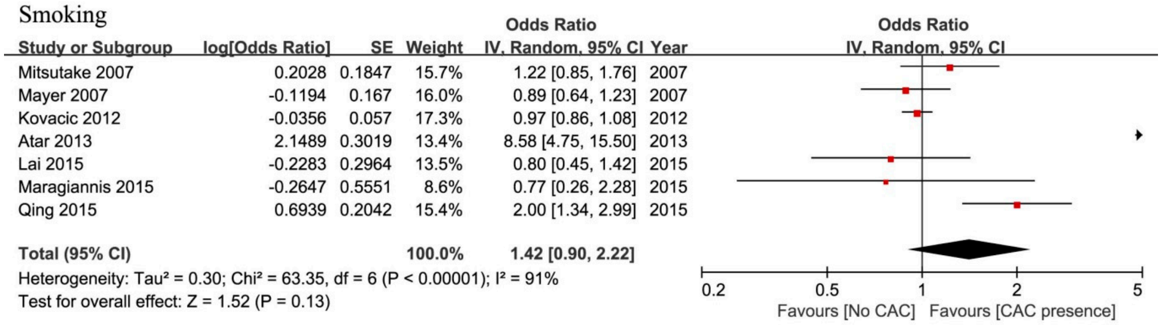

| Study or Subgroup | log[Odds Ratio] | SE | Weight | Odds Ratio IV, Random, 95% CI | Year |
|---|---|---|---|---|---|
| Mitsutake 2007 | 0.2028 | 0.1847 | 15.7% | 1.22 [0.85, 1.76] | 2007 |
| Mayer 2007 | -0.1194 | 0.167 | 16.0% | 0.89 [0.64, 1.23] | 2007 |
| Kovacic 2012 | -0.0356 | 0.057 | 17.3% | 0.97 [0.86, 1.08] | 2012 |
| Atar 2013 | 2.1489 | 0.3019 | 13.4% | 8.58 [4.75, 15.50] | 2013 |
| Lai 2015 | -0.2283 | 0.2964 | 13.5% | 0.80 [0.45, 1.42] | 2015 |
| Maragiannis 2015 | -0.2647 | 0.5551 | 8.6% | 0.77 [0.26, 2.28] | 2015 |
| Qing 2015 | 0.6939 | 0.2042 | 15.4% | 2.00 [1.34, 2.99] | 2015 |
| **Total (95% CI)** | | | **100.0%** | **1.42 [0.90, 2.22]** | |

Heterogeneity: Tau² = 0.30; Chi² = 63.35, df = 6 (P < 0.00001); I² = 91%
Test for overall effect: Z = 1.52 (P = 0.13)



Dyslipidemia

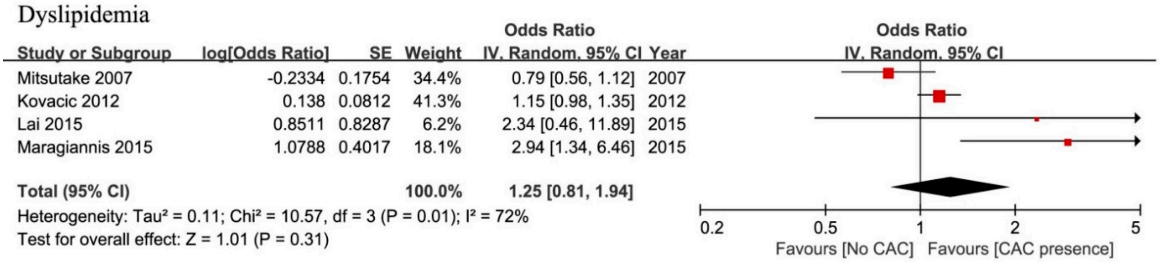

| Study or Subgroup | log[Odds Ratio] | SE | Weight | Odds Ratio IV, Random, 95% CI | Year |
|---|---|---|---|---|---|
| Mitsutake 2007 | -0.2334 | 0.1754 | 34.4% | 0.79 [0.56, 1.12] | 2007 |
| Kovacic 2012 | 0.138 | 0.0812 | 41.3% | 1.15 [0.98, 1.35] | 2012 |
| Lai 2015 | 0.8511 | 0.8287 | 6.2% | 2.34 [0.46, 11.89] | 2015 |
| Maragiannis 2015 | 1.0788 | 0.4017 | 18.1% | 2.94 [1.34, 6.46] | 2015 |
| **Total (95% CI)** | | | **100.0%** | **1.25 [0.81, 1.94]** | |

Heterogeneity: Tau² = 0.11; Chi² = 10.57, df = 3 (P = 0.01); I² = 72%
Test for overall effect: Z = 1.01 (P = 0.31)



**Figure S1.** Forest Plots. Age (OR = 1.07, $p$ = 0.04), male gender (OR = 1.47, $p$ = 0.02), hypertension (OR = 1.71, $p$ < 0.00001), and diabetes (OR = 1.34, $p$ = 0.03) were predictive for CAC presence. Smoking and dyslipidaemia were not predictive.

Male gender

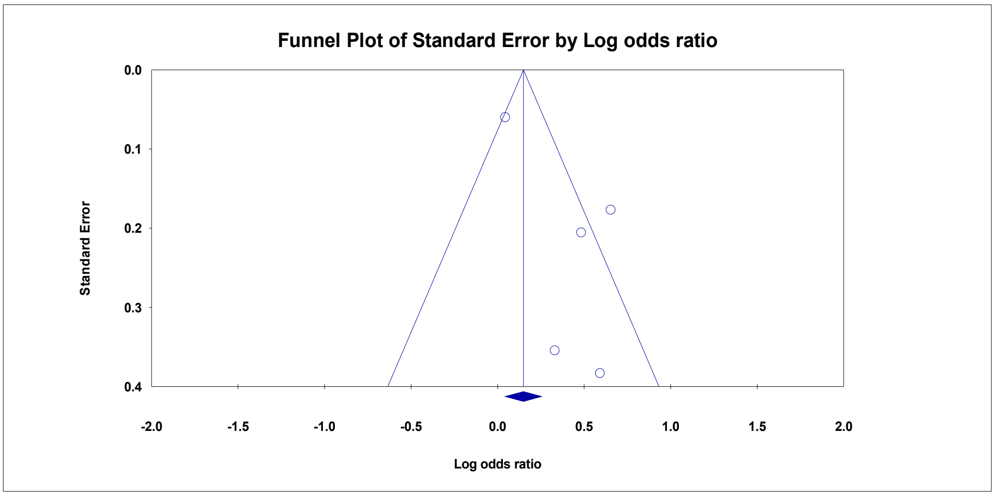


**Figure S2.** *Cont.*

Hypertension

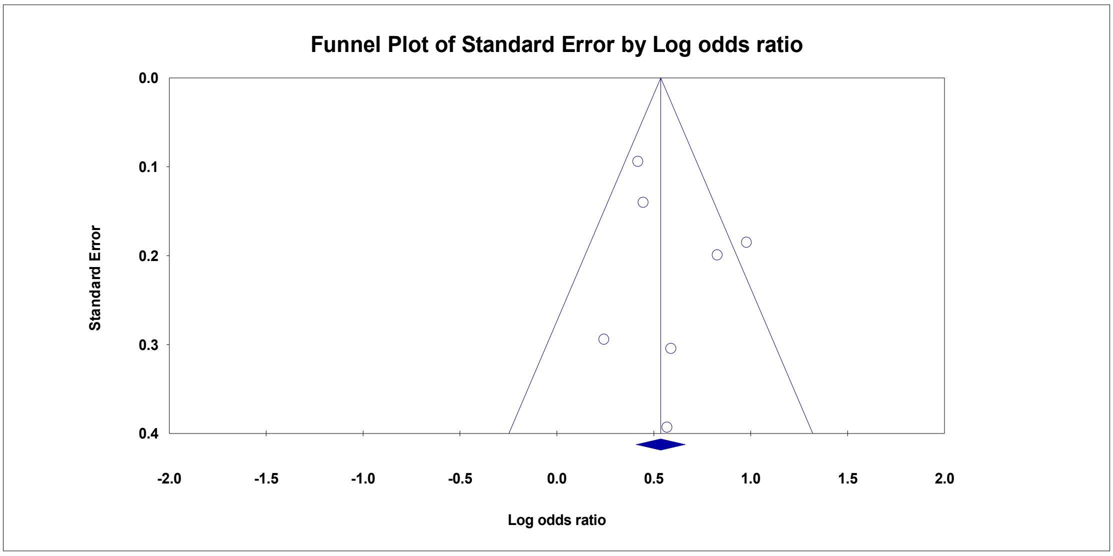


Diabetes

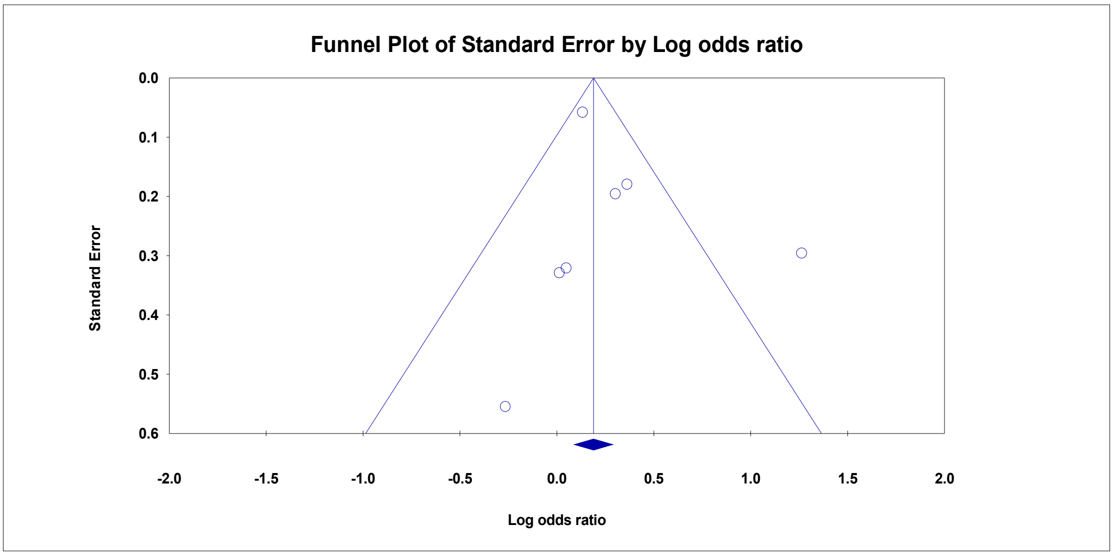


Smoking

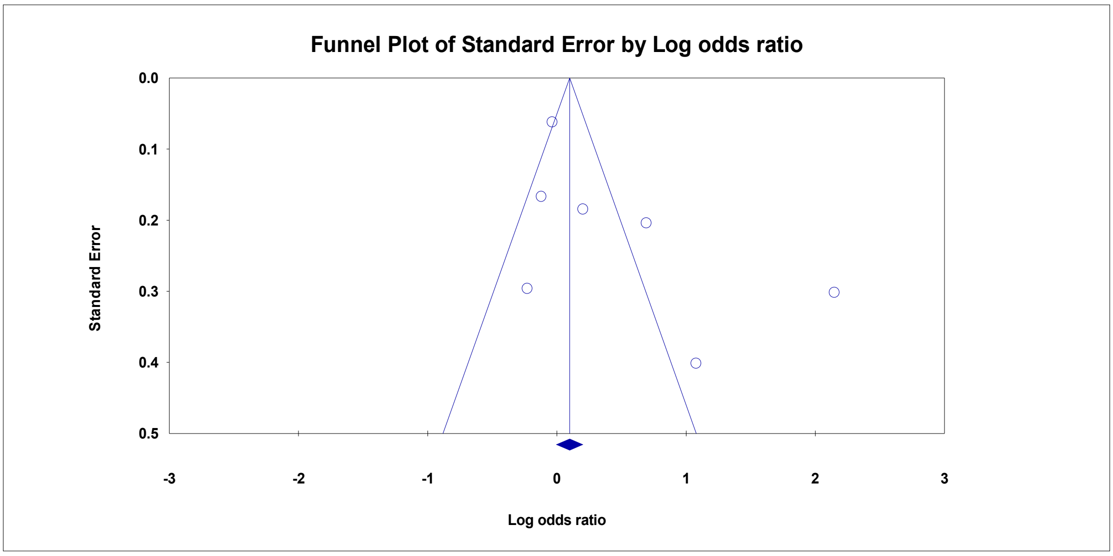


**Figure S2.** *Cont.*

Dyslipidaemia

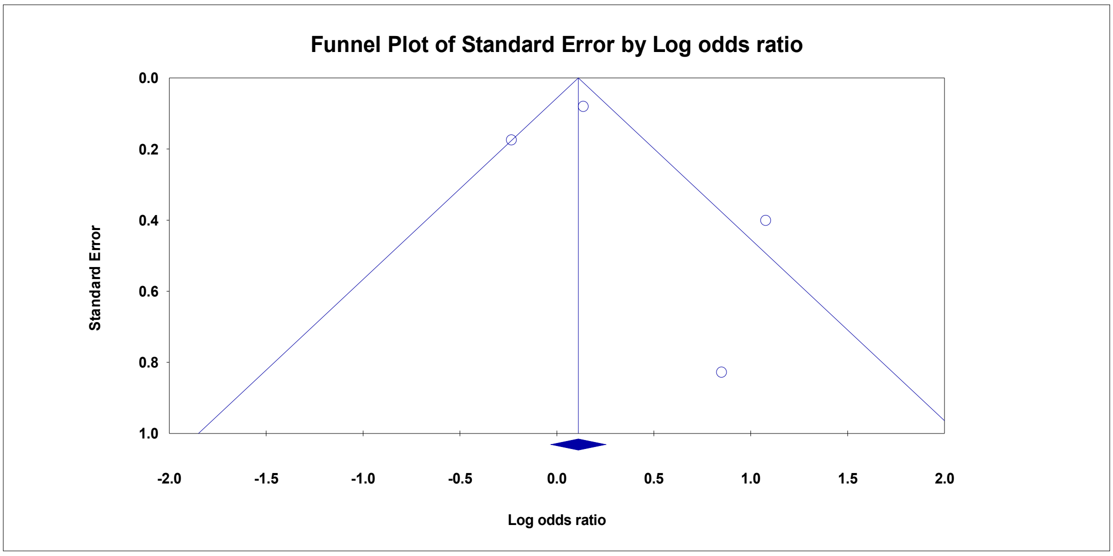


**Figure S2.** Funnel Plots.
